# The association of perirenal adipose tissue accumulation with left ventricular hypertrophy and the mediating role of insulin resistance: a cross-sectional study involving 1112 individuals with type 2 diabetes mellitus

**DOI:** 10.3389/fendo.2024.1465577

**Published:** 2025-01-09

**Authors:** Wei Wang, Yang Chen, Xiu Ping Qiu, Xiu Li Guo

**Affiliations:** Department of Endocrinology, Longyan First Affiliated Hospital of Fujian Medical University, Longyan, Fujian, China

**Keywords:** perirenal adipose tissue, left ventricular hypertrophy, insulin resistance, perirenal fat thickness, triglyceride to high-density lipoprotein cholesterol ratio

## Abstract

**Objective:**

Recent studies have underscored the metabolic and cardiovascular regulatory capacity of perirenal adipose tissue (PAT), implicating its potential involvement in the pathogenesis of left ventricular hypertrophy (LVH). This investigation aims to assess the relationship between increased PAT mass and LVH, while also examining the potential mediating role of insulin resistance in this relationship among individuals with type 2 diabetes mellitus (T2DM).

**Method:**

1112 individuals with T2DM were prospectively recruited for this study. Perirenal fat thickness (PrFT), measured using unenhanced abdominal CT, served as a measure of PAT mass. The triglyceride to high-density lipoprotein cholesterol ratio (TG/HDL-c) was computed to assess insulin resistance. LVH was identified as left ventricular mass index (LVMI) >115 g/m² in men or LVMI >95 g/m² in women. The correlations of LVH risk with PrFT and TG/HDL-c were analyzed by weighted binomial logistic regression and restricted cubic splines (RCS) analyses. Furthermore, the mediating role of TG/HDL-c in this relationship was explored using the adjusted mediation analysis.

**Results:**

Participants in the LVH group displayed significantly higher PrFT and TG/HDL-c than the non-LVH group (*P* < 0.001). Adjusting for confounding factors, the LVMI demonstrated a positive correlation with PrFT (*β*=0.262, *P*<0.001) and TG/HDL-c (*β*=0.206, *P*<0.001). PrFT and TG/HDL-c emerged as independent variables for LVH, with odds ratios of 1.33 (95%CI:1.24-1.43, *P*<0.001) and 1.20 (95%CI:1.05-1.36, *P*=0.006), respectively. Each standard deviation increases in PrFT and TG/HDL-c conferred an additional 240% (*P*<0.001) and 41% (*P*=0.006) risk for LVH. A linear correlation of LVH risk with PrFT and TG/HDL-c was observed from RCS analysis (*P* for nonlinear and overall< 0.001). Moreover, TG/HDL-c mediated 13.4% of the association between PrFT and LVMI, and 8.5% between PrFT and LVH.

**Conclusion:**

Increased PAT accumulation contributes to an independent variable for LVH, with insulin resistance acting as a mediating variable in this relationship.

## Introduction

Cardiovascular disease (CVD) is the leading cause of mortality in individuals with type 2 diabetes mellitus (T2DM), encompassing heart failure and coronary atherosclerotic heart disease (1). Obesity has risen to pandemic levels and is intricately linked with increased CVD risk and mortality (2). Abnormal accumulation of visceral adipose tissue (VAT) is a hallmark of obesity, contributing significantly to the development of CVD and other metabolic disorders. Perirenal adipose tissue (PAT) surrounds the kidney and provides essential mechanical support for renal function. Histological and embryological studies have indicated that PAT shares a developmental origin with conventional VAT, suggesting similar impacts on cardiovascular health. Recent research has underscored PAT’s unique attributes, including complete lymphatic drainage and vascularization, classifying it more as an organ than mere connective tissue (3). In recent years, several clinical studies revealed close correlations between increased PAT and cardiometabolic disorders like nonalcoholic fatty liver disease (4), subclinical carotid atherosclerosis (5), and systemic calcified atherosclerosis (6), indicating that PAT accumulation may exhibit heightened metabolic activity and behave as a risk factor for CVD. The incidence of heart failure in T2DM has been observed to increase gradually, independent of coronary atherosclerotic heart disease and hypertension. Left ventricular hypertrophy (LVH) represents a compensatory adaptation involving structural changes in cardiac morphology, which increases the risk of heart failure (7). Given the potential effects of PAT on the cardiovascular system, it is crucial to investigate whether increased PAT mass serves as an independent risk factor for LVH.

Perirenal fat thickness (PrFT), measured by non-enhanced CT scans, has emerged as a validated index proficient in accurately assessing the PAT mass (8). Clinical studies have observed a positive correlation between visceral fat area (VFA) and left ventricular mass index (LVMI), establishing VFA as an independent risk factor for LVH (9). T2DM predisposes individuals to abnormal cardiac structure and function amidst the rising prevalence of obesity. Sodium-glucose cotransporter-2 inhibitors and glucagon-like peptide-1 receptor agonists are recognized for their efficacy in reducing the risk of heart failure and body weight. Recent clinical studies have indicated that liraglutide and dapagliflozin possess PAT-reducing properties (10, 11). Identifying PAT as a potential risk factor for LVH could provide opportunities for targeted interventions with cardioprotective therapies. However, limited data exist regarding the association between increased PAT and LVH risk. Previous research has demonstrated a mediating role of insulin resistance in the relationship between visceral obesity and cardiovascular function (12), as well as hypertension incidence (13). Moreover, research has shown that PrFT acts as an independent risk factor for insulin resistance (14). Despite these findings, the potential mediating role of insulin resistance in the relationship between elevated PAT and LVH has yet to be clearly established. The triglyceride to high-density lipoprotein cholesterol ratio (TG/HDL-c), a well-recognized surrogate marker of insulin resistance, has independently been associated with both cardiometabolic risk (15) and LVH (16). To address the above concerns, this study aimed to evaluate the associations among TG/HDL-c, PrFT, and LVH, and to assess whether TG/HDL-c mediates the association between PrFT and LVH.

## Methods

### Participants and study design

This study prospectively enrolled participants with T2DM who were admitted to the inpatient department of endocrinology at Longyan First Affiliated Hospital of Fujian Medical University. Ethical approval (IC-2020069) was obtained from our hospital’s Ethics Committee, adhering strictly to the principles of the Declaration of Helsinki. Participants received detailed information about the study objectives, and written consent was obtained from each participant. Several exclusion criteria were applied during participant selection. Individuals were excluded if they presented with any of the following conditions: 1) concurrent primary or secondary heart diseases that could confound cardiac assessments (e.g., hypertrophic, infiltrative, and congestive heart failure; severe liver or kidney dysfunction; abnormal thyroid function); 2) current use of specific anti-ventricular remodeling therapies; 3) renal structural abnormalities interfering with accurate measurement of PrFT (e.g., renal space-occupying lesions, history of renal or perirenal surgery, evident perirenal or renal infection); 4) inability to undergo computed tomography scans and ultrasound assessments due to special circumstances (e.g., pregnancy, known allergy to contrast agents); 5) extreme hypertriglyceridemia and treatment with medications affecting lipid metabolism (e.g., estrogens, tamoxifen, glucocorticoids, medications for hyperlipidemic pancreatitis); 6) incomplete data. Recruitment spanned from January 2022 to May 2024. The final analysis included 1112 participants aged 29 to 74 years, with 128 participants excluded. According to the LVH statutes, participants were categorized into the LVH group (n=286) and non-LVH group (n=826).

### Exposure variable assessment

PrFT was the primary exposure variable in this study. Participants underwent unenhanced abdominal CT scans to capture detailed kidney and perirenal structural characteristics. Adipose tissue (AT) was identified based on density criteria (window center: -100 Hounsfield Units; width: -50 to -200 Hounsfield Units), distinguishing it from surrounding tissues. PAT was subsequently defined as AT from the kidney to the nearest visceral or muscle structure. Finally, PrFT was quantified as the mean maximum distance of PAT from the posterior wall of the kidney along the plane delineated by the left and right renal veins to the inner margin of the abdominal wall (17).

### Outcome variable assessment

LVH and LVMI served as the main outcome variables. Participants underwent transthoracic echocardiographic examination using a Philips EPIQ5 cardiography system (Philips Healthcare, Amsterdam, Netherlands). The assessment was conducted by experienced echocardiologists with participants positioned in the lateral decubitus posture. Cardiac structural parameters, including interventricular septum diastole (IVSd), posterior wall diastole (PWd), and left ventricular diastolic diameter (LVDd), were assessed using M-mode echocardiography, following established guidelines. LVMI was calculated using previously described formulas (18): LVMI = left ventricular mass (LVM) divided by body surface area (BSA), where LVM was calculated as 0.80 × 1.04 × {(IVSd + LVDd + PWd)³ - LVDd³} + 0.6 g. BSA was computed using the Du Bois formula (19): BSA=0.007184 × weight (kg)^0.425^ × height (cm)^0.725^. LVH was identified as LVMI >115 g/m² in men or LVMI >95 g/m² in women (18).

### Assessment of mediator and study covariates

TG/HDL-c was the mediator, indicative of insulin resistance. The TG/HDL-c was calculated using previously described formulas: TG/HDL-c = serum TG/HDL cholesterol level. Study covariates encompassed age, gender, diabetic duration, smoking, hypertension, systolic blood pressure (SBP) diastolic blood pressure (DBP), glycated hemoglobin A1c (HbA1c), creatinine, TG, total cholesterol (TC), low-density lipoprotein cholesterol (LDL-c), HDL-c, uric acid (UA), body mass index (BMI), waist circumference, VFA, and subcutaneous fat area (SFA). Trained interviewers collected demographic information, current or prior medication use, and any pertinent medical histories meeting exclusion criteria. Trained research nurses employed standardized methods to measure various physical examination parameters. BMI was calculated as weight (kg) divided by the square of height (m²). Serum biochemical markers were analyzed using automatic electrochemiluminescence immunoassay analyzers (Roche Diagnostics Corporation), and HbA1c levels were assessed using high-performance liquid chromatography. VFA and SFA were measured by trained operators using a dual bioelectrical impedance analyzer (DUALSCANHDS-2000, Omron Healthcare Company, Japan).

### Statistical analysis

Data storage and statistical analyses were performed using SPSS version 26.0 or R version 4.2.3 software. Baseline characteristics of the study cohort were expressed as means ± standard deviation (SD) or frequency tables (N, %). Student’s t-test or the chi-square test was utilized to compare baseline characteristics between the LVH and non-LVH groups. Spearman correlation analysis assessed the relationships among PrFT, TG/HDL-c, and LVMI. These correlations were further examined using weighted multiple regression analysis, adjusting for confounding factors across three models. Model 1 adjusted for age, gender, duration of diabetes, and smoking status. Model 2 additionally adjusted for metabolic profiles like hypertension, HbA1c, TG, TC, LDL-c, HDL-c, and UA. Model 3 further included adjusting obesity-related indexes like BMI, WC, VFA, and SFA. Weighted binomial logistic regression and restricted cubic splines (RCS) analysis were conducted to investigate the correlations of LVH risk with PrFT and TG/HDL-c. Adjusted mediation models using bootstrapping methods evaluated the mediating role of TG/HDL-c in these associations. Sensitivity analyses excluding participants with hypertension were conducted to validate these findings further. Statistical significance was set at *P* < 0.05 (two-tailed).

## Result

### Baseline characteristics of the participants based on LVH statutes

In the final analysis, 1112 individuals with T2DM, characterized by an average diabetic duration of 8.4 ± 3.4 years and an age range of 30 to 74 years, were included. The study population consisted of 286 individuals (25.7%) with LVH and 826 individuals (74.3%) without LVH. A comparison of clinical characteristics based on LVH status is outlined in **Table 1**. Significant differences were observed between participants in the LVH group and those in the non-LVH group. The LVH group demonstrated advanced age and longer diabetes duration (*P*<0.001). Moreover, individuals with LVH displayed elevated levels of metabolic parameters such as TG, SBP, DBP, and UA, as well as obesity-related indices including BMI, WC, SFA, and VFA, alongside decreased levels of HDL-c (*P*<0.001). The prevalence of hypertension was also notably higher among LVH participants (*P*<0.001). Furthermore, the LVH group exhibited considerably higher PrFT and TG/HDL-c when compared with the non-LVH group (*P* < 0.001).

**Table 1:** Comparison of baseline characteristics between LVH and non-LVH groups.

Characteristics	Total (n=1112)	LVH (n=286)	Non-LVH (n=826)	*P* value
Age (year)	52.9 ± 8.1	54.9 ± 7.9	52.2 ± 8.1	<0.001
Male, n (%)	578 (52.0)	157 (54.9)	421 (51.0)	0.252
Diabetic Duration (year)	8.4 ± 3.4	9.2 ± 3.5	8.0 ± 3.2	<0.001
BMI (kg/m^2^)	24.5 ± 3.0	26.1 ± 3.3	24.0 ± 2.8	<0.001
WC (cm)	85.9 ± 7.1	89.5 ± 7.9	84.7 ± 6.3	<0.001
SBP (mmHg)	133.6 ± 17.8	144.5 ± 14.2	129.8 ± 17.5	<0.001
DBP (mmHg)	81.5 ± 9.2	86.1 ± 8.2	79.9 ± 9.0	<0.001
HbA1c (%)	8.7 ± 1.0	8.7 ± 1.1	8.7 ± 0.9	0.750
TG (mmol/L)	2.21 ± 1.40	3.20 ± 1.72	1.87 ± 1.08	<0.001
TC (mmol/L)	5.20 ± 1.27	5.28 ± 1.34	5.14 ± 1.23	0.206
HDL-c(mmol/L)	1.09 ± 0.24	0.99 ± 0.20	1.13 ± 0.23	<0.001
LDL-c(mmol/L)	3.56 ± 0.96	3.47 ± 0.96	3.59 ± 0.97	0.060
UA (umol/L)	358.9 ± 86.5	396.8 ± 84.2	345.8 ± 83.4	<0.001
Creatinine (umol/L)	68.8 ± 13.2	69.4 ± 13.4	68.6 ± 13.2	0.377
VFA (cm^2^)	85.9 ± 23.5	97.5 ± 20.6	83.0 ± 23.8	<0.001
SFA (cm^2^)	182.6 ± 43.3	194.3 ± 43.8	178.5 ± 41.3	<0.001
PrFT (mm)	13.0 ± 4.7	16.5 ± 3.8	11.7 ± 4.3	<0.001
TG/HDL-c	2.31 ± 1.90	3.60 ± 2.45	1.87 ± 1.41	<0.001
Hypertension, n (%)	391 (35.2)	172 (60.1)	219 (26.5)	<0.001
Smoking, n (%)	410 (36.9)	111 (38.8)	299 (36.2)	0.430

Data were expressed as means ± standard deviation or frequency tables (N, %).

BMI, body mass index; WC, waist circumference; HbA1c, glycated hemoglobin; UA, uric acid; TG, triglyceride; TC, total cholesterol; HDL-c, high-density lipoprotein cholesterol; LDL-c, low-density lipoprotein cholesterol; SBP, systolic blood pressure; DBP, diastolic blood pressure; VFA, visceral fat area; SFA, subcutaneous fat area; PrFT, perirenal fat thickness; TG/HDL-c, triglyceride to high-density lipoprotein cholesterol ratio; LVH, center ventricular hypertrophy.

### Associations among PrFT, TG/HDL-c, and LVMI

**Figure 1:**
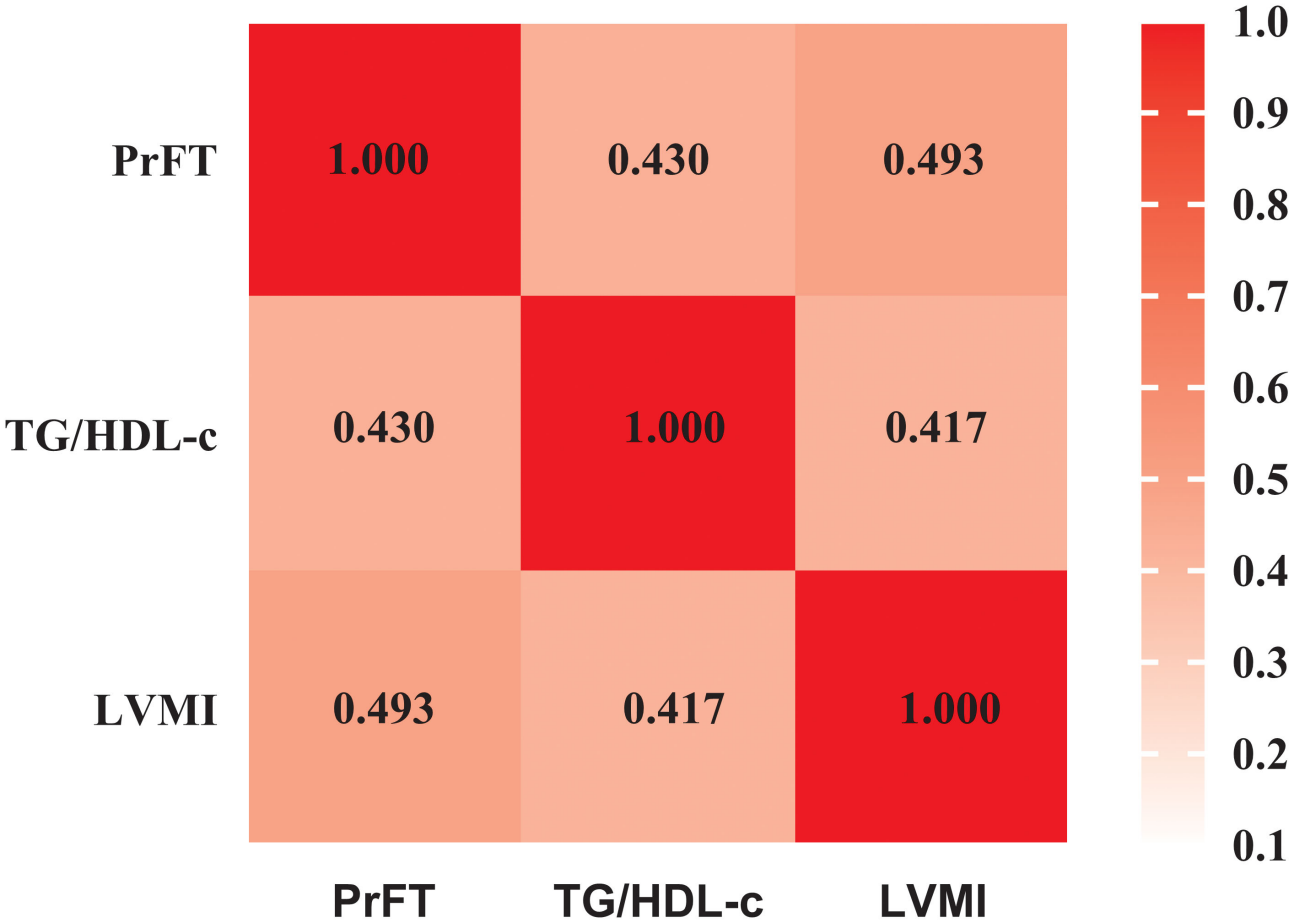
Pearson correlation analysis examining the relationships among PrFT, TG/HDL-c, and LVMI. PrFT: Perirenal fat thickness. TG/HDL-c: Triglyceride to high-density lipoprotein cholesterol ratio. LVMI: Left ventricular mass index.

**Figure 1** displays the relationships among PrFT, TG/HDL-c, and LVMI as examined through the Spearmen or Pearson correlation analysis. LVMI exhibited a positive correlation with PrFT (*r*= 0.493, *P* < 0.001) and TG/HDL-c (*r*= 0.417, *P* < 0.001). Additionally, PrFT showed a positive association with TG/HDL-c (*r*= 0.430, *P* < 0.001). Subsequently, these correlations were further analyzed by the weighted multiple linear regression analysis after adjusting for three models (**Table 2**). In both Model 1 and Model 2, PrFT and TG/HDL-c showed positive correlations with LVMI (*P* < 0.001), along with a positive association between PrFT and TG/HDL-c (*P* < 0.001). Notably, following adjustments for obesity-related indexes in Model 3, the positive correlations of LVMI with PrFT (*β*=0.262, *P*<0.001) and TG/HDL-c (*β*=0.206, *P*<0.001) persisted. Additionally, PrFT revealed a positive association with TG/HDL-c (*β*=0.171, *P*<0.001).

**Table 2:** Weighted multivariate linear regression analysis for the independent associations among PrFT, TG/HDL-c, and LVMI.

Independent variables	Dependent variables	Model 1		Model 2		Model 3	
		*β*	*P*	*β*	*P*	*β*	*P*
PrFT	LVMI	0.413	<0.001	0.387	<0.001	0.262	<0.001
TG/HDL-c	LVMI	0.394	<0.001	0.346	<0.001	0.206	<0.001
TG/HDL-c	PrFT	0.318	<0.001	0.277	<0.001	0.171	<0.001

Model 1: adjusted for age, gender, diabetic duration, smoking.

Model 2: adjusted for hypertension, glycated hemoglobin A1c, triglyceride, total cholesterol, low-density lipoprotein cholesterol, high-density lipoprotein cholesterol, and uric acid based on Model 1.

Model 3: additionally adjusted for body mass index, waist circumference, visceral fat area, and subcutaneous fat area based on Model 1 and Model 2.

PrFT, perirenal fat thickness; TG/HDL-c, triglyceride to high-density lipoprotein cholesterol ratio; LVMI, center ventricular mass index.

### Correlations of LVH risk with PrFT and TG/HDL-c

**Figure 2:**
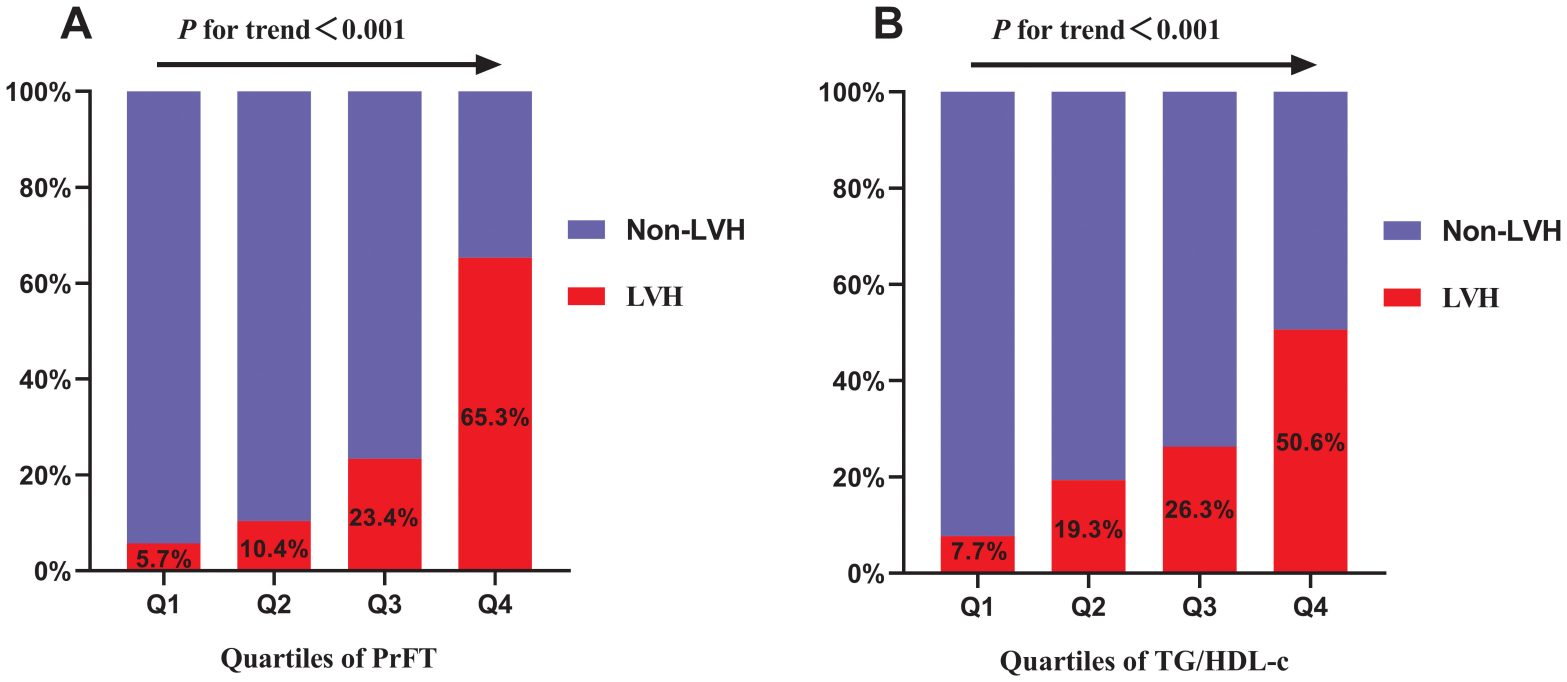
Prevalence of LVH across the quartiles of PrFT **(A)** and TG/HDL-c **(B)**. Perirenal fat thickness. TG/HDL-c: Triglyceride to high-density lipoprotein cholesterol ratio. LVH: Left ventricular hypertrophy.

**Figure 3:**
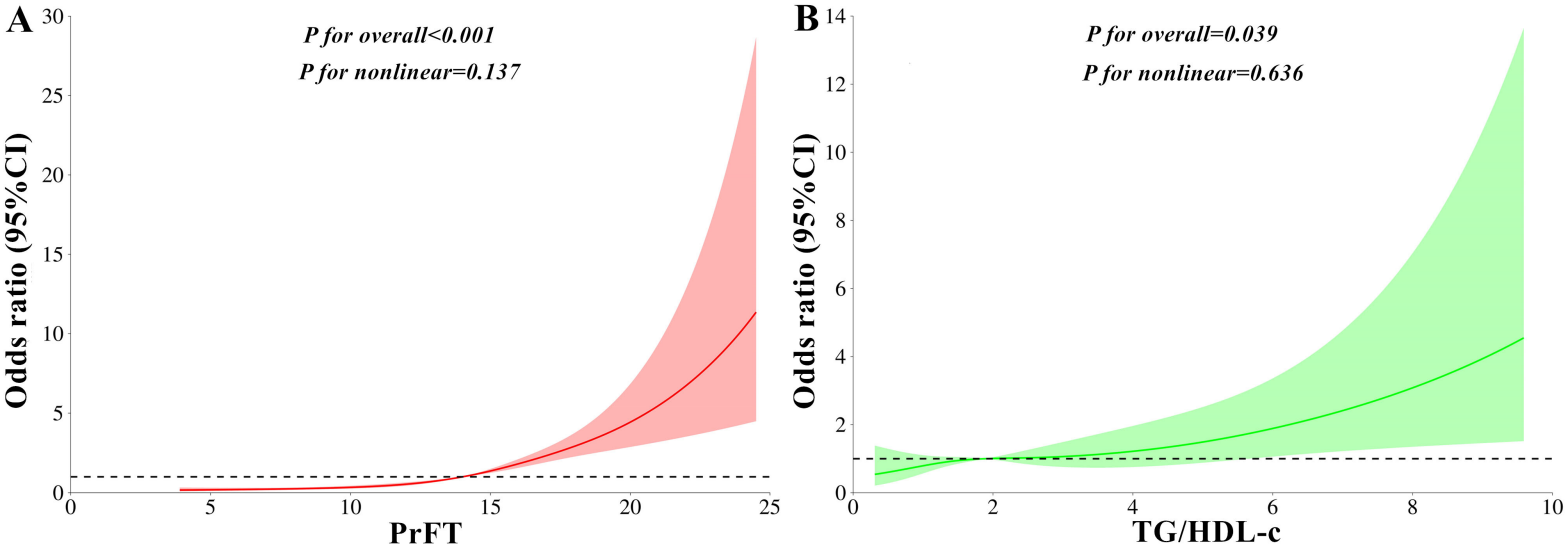
Linear correlations of LVH risk with PrFT **(A)** and TG/HDL-c **(B)** after adjusting for Model 3 as analyzed by the restricted cubic splines analysis. Model 3 adjusted for age, gender, diabetic duration, smoking, metabolic profiles like hypertension, glycated hemoglobin A1c, triglyceride, total cholesterol, low-density lipoprotein cholesterol, high-density lipoprotein cholesterol, uric acid, obesity-related indexes like body mass index, waist circumference, visceral fat area, and subcutaneous fat area. PrFT: perirenal fat thickness. TG/HDL-c: triglyceride to high-density lipoprotein cholesterol ratio. LVH: left ventricular hypertrophy.

**Figure 2** displays the prevalence of LVH across quartiles of PrFT and TG/HDL-c. Substantial increases in LVH prevalence were observed across both higher PrFT (**Figure 2A**) and TG/HDL-c (**Figure 2B**) quartiles compared to lower quartiles (*P*<0.001). A weighted binomial logistic regression was employed to assess the association of LVH risk with PrFT and TG/HDL-c after adjusting for confounding factors in three models, as shown in **Table 3**. Results indicated that higher PrFT and TG/HDL-c quartiles exhibited positive correlations with LVH risk across all three models (*P*<0.05). In Model 3, following full adjustment for confounding factors, PrFT and MHR emerged as independent variables for LVH risk, with odds ratios of 1.33 (95%CI:1.24-1.43, *P*<0.001) and 1.20 (95%CI:1.05-1.36, *P*=0.006) respectively. Furthermore, each SD increase in PrFT and TG/HDL-c revealed an additional 240% (*P*<0.001) and 41% (*P*=0.006) risk for LVH, indicating a significant dose-response relationship between PrFT, TG/HDL-c, and LVH risk (*P*<0.001). As shown in **Figure 3**, the RCS analysis also identified a linear association between PrFT and LVH risk (*P* for overall<0.001; *P* nonlinear=0.137), as well as TG/HDL-c and LVH risk (*P* for overall=0.039; *P* nonlinear=0.636).

**Table 3:** Weighted binomial logistic regression analysis for the correlations of LVH risk with PrFT and TG/HDL-c.

Variable	Model 1		Model 2		Model 3	
	OR (95%CI)	*P* value	OR (95%CI)	*P* value	OR (95%CI)	*P* value
PrFT (mm)						
Per SD increase	4.54(3.55-5.81)	<0.001	4.23(3.23-5.54)	<0.001	3.40(2.47-4.68)	<0.001
Overall	1.39(1.31-1.46)	<0.001	1.36(1.29-1.46)	<0.001	1.33(1.24-1.43)	<0.001
Q1	Ref. (1.0)		Ref. (1.0)		Ref. (1.0)	
Q2	1.52(1.08-2.15)	0.018	1.46(1.16-1.98)	0.006	1.13(1.10-1.16)	<0.001
Q3	4.40(2.46-7.88)	<0.001	4.30(2.29-8.08)	<0.001	3.09(1.49-6.40)	<0.001
Q4	9.53(6.64-13.40)	<0.001	7.40(5.30-10.73)	<0.001	4.15(2.35-7.49)	<0.001
*P* for trend	<0.001		<0.001		<0.001	
TG/HDL-c						
Per SD increase	2.45(2.09-2.87)	<0.001	`2.08(1.74-2.49)	<0.001	1.41(1.11-1.80)	0.006
Overall	1.60(1.47-1.75)	<0.001	1.47(1.34-1.62)	<0.001	1.20(1.05-1.36)	0.004
Q1	Ref. (1.0)		Ref. (1.0)		Ref. (1.0)	
Q2	2.93(1.71-5.03)	<0.001	2.48(1.42-4.32)	0.001	2.28(1.30-4.01)	0.004
Q3	4.21(2.51-7.06)	<0.001	2.98(1.69-5.25)	0.006	2.60(1.46-4.63)	0.001
Q4	6.78(4.10-10.56)	<0.001	4.05(1.93-8.67)	<0.001	3.01(1.63-5.51)	<0.001
*P* for trend	<0.001		<0.001		<0.001	

Model 1: adjusted for age, gender, diabetic duration, and smoking.

Model 2: adjusted for hypertension, glycated hemoglobin A1c, triglyceride, total cholesterol, low-density lipoprotein cholesterol, high-density lipoprotein cholesterol, and uric acid based on Model 1.

Model 3: additionally adjusted for body mass index, waist circumference, visceral fat area, and subcutaneous fat area based on Model 1 and Model 2.

PrFT, perirenal fat thickness; TG/HDL-c, triglyceride to high-density lipoprotein cholesterol ratio; LVH, center ventricular hypertrophy.

### The mediating role of TG/HDL-c

**Figure 4:**
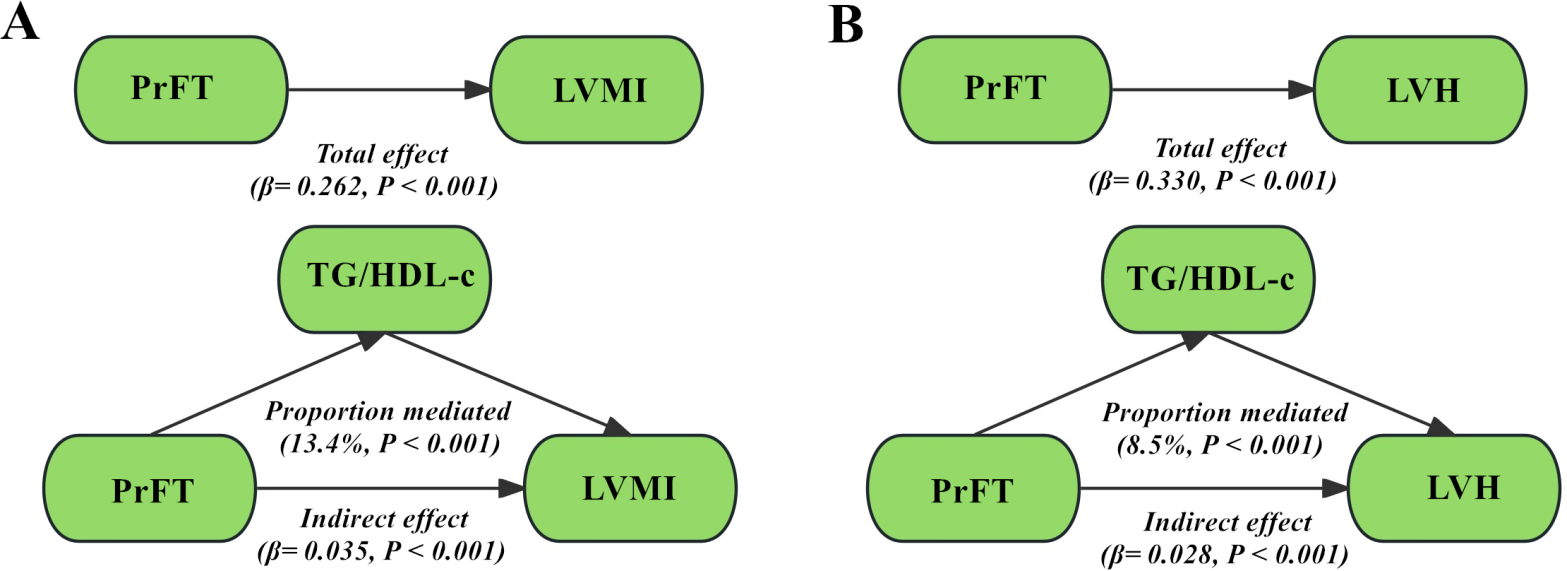
Adjusted mediation models using bootstrapping methods evaluated the mediating role of TG/HDL-c in the correlations of PrFT with LVMI **(A)** and LVH **(B)** in Model 3. PrFT: perirenal fat thickness. TG/HDL-c: triglyceride to high-density lipoprotein cholesterol ratio. LVH: left ventricular hypertrophy. LVMI: Left ventricular mass index.

**Figure 4** illustrates the structural models elucidating the mediating role of TG/HDL-c in the relationship between PrFT and LVMI, as well as PrFT and LVH risk, after comprehensive adjustments in Model 3 as evaluated through adjusted mediation analysis. The total effect of PrFT on LVMI was determined to be 0.262, with an indirect effect of 0.035. TG/HDL-c mediated 13.4% of this association (**Figure 4A**). Additionally, the total effect of PrFT on LVMI was calculated as 0.330, with an indirect effect of 0.028, corresponding to a mediated proportion of 8.5% attributable to TG/HDL-c (**Figure 4B**).

### Sensitivity analysis

To further validate the aforementioned findings, sensitivity analyses were performed, excluding participants with hypertension. Following comprehensive adjustments in Model 3, LVMI maintained a significant positive correlation with PrFT (*β*=0.225, *P*<0.001) and TG/HDL-c (*β*=0.189, *P*<0.001). The positive association between HDL-c and PrFT (β=0.145, P<0.001) persisted. Moreover, both PrFT and TG/HDL-c independently correlated with LVH, with ORs of 1.31 (95% CI: 1.19-1.44, *P*<0.001) and 1.28 (95% CI: 1.03-1.59, *P*=0.025) respectively. Each SD increase in PrFT and TG/HDL-c conferred an additional 245% (*P*<0.001) and 60% (*P*=0.026) risk for LVH. As illustrated in **Supplementary Figure 1**, TG/HDL-c mediated 12.4% of the association between PrFT and LVMI, and 11.7% of the association between PrFT and LVH.

## Discussion

Obesity is intricately linked to progressive cardiac structural and functional changes, often resulting in LVH and potentially advancing to heart failure. While increased PAT is well-established as a risk factor for cardiometabolic disorders, its association with LVH remains underexplored. This study evaluated the association between PrFT and LVH and assessed whether TG/HDL-c mediates this association in T2DM. Several significant findings emerged from this investigation. Firstly, weighted multiple linear regression analysis demonstrated positive correlations among PrFT, TG/HDL-c, and LVMI. Secondly, the weighted binomial logistic regression and RCS analysis showed that PrFT and TG/HDL-c contributed to an independent risk factor for LVH. Thirdly, TG/HDL-c partially mediated the correlations of PrFT with LVMI and LVH. Lastly, Sensitivity analyses excluding participants with hypertension further validated these associations.

T2DM tends to be associated with various metabolic disorders, making it prone to be accompanied by LVH (20). Given its frequent occurrence and potential progression to heart failure, current guidelines underscore the critical need for early detection and management of LVH and its associated risk factors to prevent its onset or advancement to heart failure (21). Previous research has identified age, UA levels, hyperlipidemia, obesity, hypertension, and insulin resistance as significant risk factors for LVH or CVD (22–24). Therefore, our study found that the LVH group exhibited advanced age, longer duration of diabetes, elevated levels of metabolic parameters such as TG, SBP, DBP, UA, and the TG/HDL-c, as well as indices related to obesity including BMI, WC, SFA, VFA, and PrFT. As we all know, obesity plays a pivotal role in the early pathogenesis of CVD. Moreover, large-scale population-based cohort studies have elucidated that the distribution of body fat profoundly modulates the impact of obesity on CVD and LVH (25–27). PAT, which shares developmental origins with VAT, fulfills analogous cardiovascular roles. Situated adjacent to the kidneys in the retroperitoneal space, PAT exhibits distinctive characteristics, including comprehensive lymphatic drainage, vascularization, innervation, and specific morphological features such as fascial borders and an independent sympathetic architecture. These unique attributes suggest that PAT functions more akin to an organ than mere connective tissue, potentially providing a structural foundation for its regulatory role in cardiovascular and metabolism homeostasis through neural, humoral, and kidney-mediated mechanisms (3, 28). While several observational studies have demonstrated an independent association between increased PAT and various cardiometabolic disorders such as metabolic syndrome (29), nonalcoholic fatty liver disease (30), and subclinical carotid atherosclerosis (5), data on the association between increased PAT and LVH remain limited. Previous studies have established that abnormal accumulation of VAT, epicardial adipose tissue (EAT), and pericardial adipose tissue independently increases the risk of LVH, regardless of traditional risk factors for LVH (9, 31, 32). Our study corroborates these findings, demonstrating a positive correlation between PrFT and LVMI, establishing PrFT as an independent predictor of LVH. Importantly, we adjusted for traditional LVH risk factors and further controlled for VFA and SFA to mitigate the potential confounding effects of VAT and subcutaneous adipose tissue. This approach distinguishes our investigation from previous studies that primarily focused on the association of LVH with EAT and pericardial adipose tissue thickness. Additionally, the sensitivity analyses further validated the above findings after excluding participants with hypertension. These results suggest that increased PAT serves as an independent risk factor for LVH, beyond the influence of traditional risk factors and VAT in LVH pathogenesis. Cuatrecasas et al. revealed that both Metformin and Dapagliflozin promote fat loss in layers associated with metabolic syndrome, with the combined treatment showing particularly effective results in reducing PAT (10). Additionally, another study reported that Liraglutide induces greater fat loss in the layers involved with metabolic syndrome, with the greatest reduction observed in PAT (11). Future studies are warranted to determine whether these medications can prevent the development of LVH and delay its progression by reducing PAT.

The intricate mechanism underlying PAT’s role in LVH may involve its multifaceted effects on the cardiovascular and endocrine systems. Previous studies have demonstrated significant correlations between PrFT and both TG and HDL-c. Ke et al. reported a negative correlation between PrFT and serum HDL-c levels in patients with T2DM (33). At the same time, D’Marco et al. identified a positive association between PrFT and serum TG levels in a cohort of 103 patients with chronic kidney disease (34). TG/HDL-c was a convenient surrogate marker for insulin resistance and was widely used to evaluate the association between insulin resistance and cardiometabolic disorders. This study revealed that TG/HDL-c is positively correlated with PrFT and acts as a risk factor for LVH, suggesting that insulin resistance may play a role in the relationship between PrFT and LVH risk. Several clinical studies have observed close correlations between increased PrFT and other traditional risk factors for LVH. De Pergola et al. observed that PrFT was positively associated with mean 24-hour DBP levels in overweight and obese subjects, implying a potential direct role of PAT in raising daily DBP (35). Ricci et al. found that the need for antihypertensive medications and the reduction in SBP were independently associated with the decrease in PAT among 89 hypertensive obese patients who underwent sleeve gastrectomy (36). Yang, Y et al. and Lamacchia, O et al. also found a positive association between PrFT and serum UA levels in T2DM (37, 38). Given the multiple mechanisms underlying the association between PAT accumulation and LVH development, elucidating the precise role of insulin resistance presents a significant challenge. This study employed mediation analysis, adjusting for potential confounding variables, to evaluate the involvement of insulin resistance in this relationship. The results showed that TG/HDL-c partially mediated the correlations of PrFT with LVMI and LVH, underscoring the pivotal role of insulin resistance in the association between increased PAT accumulation and LVH development.

### Strength and limitation

This study demonstrates strength in establishing the independent association between PrFT and the risk of LVH and elucidating the mediating role of the TG/HDL-c in this relationship, a finding corroborated by sensitivity analysis. However, several limitations warrant acknowledgment. Firstly, the data utilized in this analysis were cross-sectional, thereby limiting our ability to infer causality or assess temporal relationships. Secondly, PrFT measured by CT scan provides a reliable estimate of PAT mass, benefiting from CT’s superior resolution for adipose tissue compared to ultrasound, which aids in distinguishing PAT from surrounding tissues. Nonetheless, the utility of CT scans is tempered by radiation exposure concerns, precluding their use in certain populations such as pregnant women and children. Additionally, the study population was limited to a single regional medical center, where participants likely shared similar diets, food sources, public health initiatives, and social environments. Future multicenter studies are needed to include diverse populations from various regions and ethnic backgrounds to further validate these findings.

## Conclusion

This study revealed an independent association between PrFT and LVH risk, with the TG/HDL-c ratio partially mediating this relationship. These findings underscore the significant contribution of increased PAT accumulation in LVH development, highlighting the intermediary influence of insulin resistance in this pathway. Developing strategies to manage PAT accumulation may be beneficial in preventing LVH.

## Data Availability

The original contributions presented in the study are included in the article/**Supplementary Material**. Further inquiries can be directed to the corresponding author.

## Supplementary material

Supplementary Figure 1 The mediating role of TG/HDL-c in the correlations of PrFT with LVMI **(A)** and LVH **(B)** in Model 3 excluding participants with hypertension. PrFT: perirenal fat thickness. TG/HDL-c: triglyceride to high-density lipoprotein cholesterol ratio. LVH: left ventricular hypertrophy. LVMI: Left ventricular mass index.
