# Supplementary figures and images for: The association of perirenal adipose tissue accumulation with left ventricular hypertrophy and the mediating role of insulin resistance: a cross-sectional study involving 1112 individuals with type 2 diabetes mellitus
Source: Front Endocrinol (Lausanne). 2025 Jan 9;15:1465577. doi: 10.3389/fendo.2024.1465577 (PMC11754053; doi:10.3389/fendo.2024.1465577)

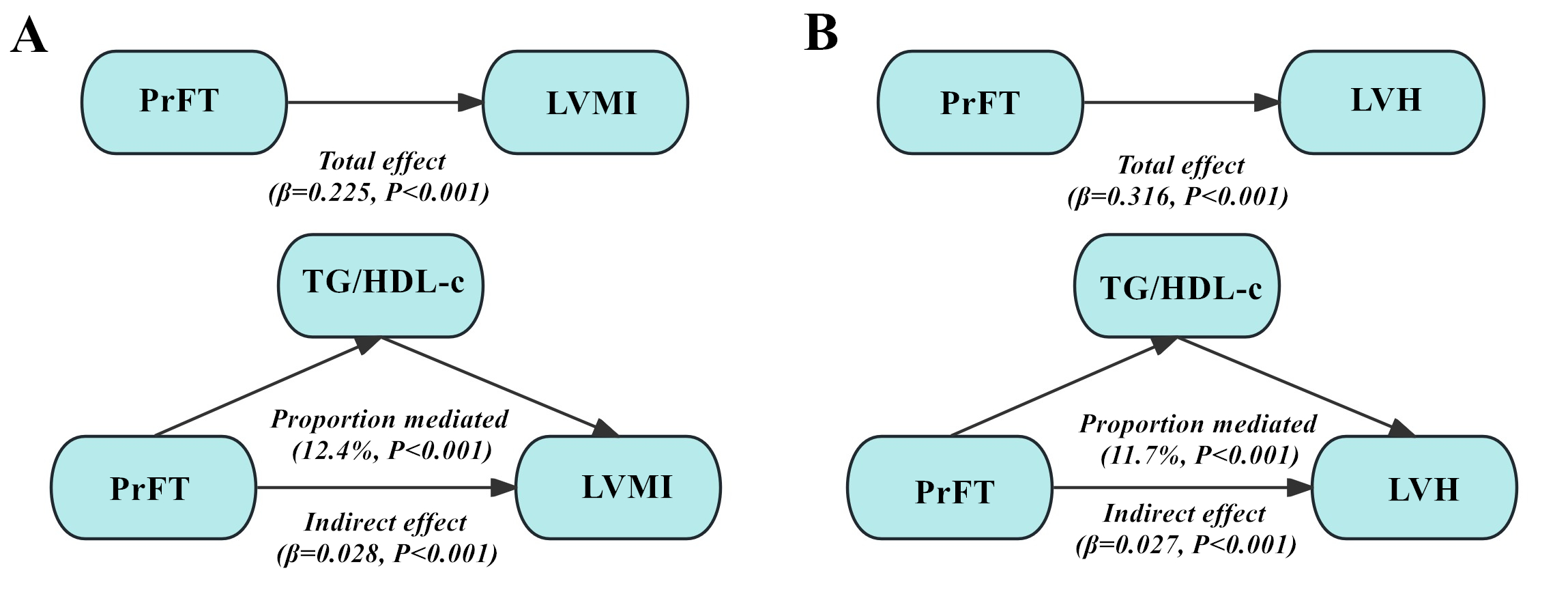

Supplement: Supplementary Figure 1 — The mediating role of TG/HDL-c in the correlations of PrFT with LVMI (A) and LVH (B) in Model 3 excluding participants with hypertension. PrFT: perirenal fat thickness. TG/HDL-c: triglyceride to high-density lipoprotein cholesterol ratio. LVH: left ventricular hypertrophy. LVMI: Left ventricular mass index. [file Image1.jpeg]
